# Osteoporosis: molecular pathogenesis and therapeutic interventions

**DOI:** 10.1186/s43556-025-00349-5

**Published:** 2025-11-05

**Authors:** Xubin Zhang, Yongsheng Liang, Fayao Zhang, Xiaoyuan Liu

**Affiliations:** 1 Department of Orthopaedic, Xi’an Hospital of Traditional Chinese Medicine, No. 69 Fengcheng 8 Road, Xi’an, Shaanxi 710016 China; 2 Department of Nephrology, Shaanxi Provincial Hospital of Traditional Chinese Medicine, No. 4 Xihuamen, Xi’an, Shaanxi 710003 China

**Keywords:** Osteoporosis, Molecular pathogenesis, Therapeutic intervention, Research progress

## Abstract

Osteoporosis is a systemic skeletal disease. Genetic and environmental factors work together to cause increased bone resorption, decreased bone formation, bone remodeling imbalance, reduced bone mass, and increased bone fragility. The global incidence of osteoporosis is relatively high, and osteoporosis negatively affects health and quality of life. Prevention and treatment research has continuously attracted the attention of scholars worldwide, and there is an extremely urgent need to find effective and safe treatment plans. This review elaborates on the physiological structure of bones and the principal relationship between bones and osteoporosis. The molecular mechanisms of osteoporosis development, including genes, inflammation, oxidative stress, signaling pathways, intestinal microbiota, autophagy, and iron metabolism, are systematically reviewed. This review comprehensively summarizes the latest advancements in the diagnosis and therapeutic interventions for osteoporosis. The therapeutic interventions include Western medicine treatment, Chinese herbal medicine treatment, nonpharmacological management and emerging therapeutic strategies. This review explores in depth the advantages and disadvantages of Western medicine and Chinese herbal medicine treatments, highlights the challenges that Chinese herbal medicine treatment for osteoporosis must overcome, and reveals the gap between emerging treatment methods and clinical applications, as well as potential directions for osteoporosis research, aiming to provide valuable references for the treatment of osteoporosis in the future.

## Introduction

Osteoporosis (OP) is a global health challenge that severely affects survival and quality of life. OP is a metabolic bone disease, and its occurrence and development mechanism are very complex and related to a variety of factors, such as genes, immune factors, drug factors, inflammation, and age. OP can destroy the normal physiological structure of bone, leading to a reduction in human bone mass and a decrease in bone strength and a substantial increase in the risk of fracture. Data show that worldwide, more than 200 million people worldwide suffer from OP; additionally, the overall prevalence is 18.3%. In terms of global geographical distribution, people in Europe have a relatively high probability of osteoporotic fractures, affecting up to 34.8% of this population. From the perspective of sex, women, especially postmenopausal women, have a greater probability of OP than men do. A survey study revealed that the prevalence of OP among postmenopausal women in Kenya is as high as 26.4% [1–5]. From 2008 to 2018, the incidence of OP in the Korean population aged 50 years and older was 18.4 cases per 1,000 people per year [6]. Data from 2018 revealed that the prevalence rate of OP among people aged 50 years and older in China was 19.2% [7]. OP affects a large patient population worldwide and is characterized by a high prevalence rate.

OP can be divided into primary OP and secondary OP, of which primary OP is related mainly to aging factors. The most common clinical OP patients are postmenopausal women, and secondary OP stems from various causes, including endocrine diseases and drug effects [8]. Modern medical treatments for OP aim to improve bone metabolism, promote bone formation, and inhibit bone resorption. Drugs that inhibit bone resorption include estrogen, bisphosphonate, monoclonal antibodies, and calcitonin, and drugs that promote bone formation include parathyroid hormone and active vitamin D [9]. These drugs have achieved good efficacy in clinical practice, alleviating symptoms of discomfort and improving quality of life. However, these drugs also have many drawbacks, such as high cost, frequent adverse reactions, and certain carcinogenicity. In recent years, there have been numerous reports on clinical treatment with and related research on traditional Chinese medicine for treating OP. However, most studies on Chinese herbal medicines remain at the animal experiment stage, and there are many challenges with regard to translating such medicines into clinical use. At present, the prevention and treatment of OP remain a global challenge that urgently needs to be addressed.

In light of this background, This review first introduces the theoretical relationship between bone and OP and elaborates on the molecular mechanisms of OP from the aspects of genetic factors, environmental factors, signaling pathways, and bone remodeling. Next, the article summarizes the progress in the diagnosis of OP and comprehensively reviews the treatment regimens for OP. Treatment regimens include Western medicine treatment, Chinese herbal medicine treatment, nonpharmacological management and emerging therapeutic strategies. This review focuses on the molecular mechanisms and therapeutic progress of OP; it explores in depth the advantages and disadvantages of Western medicine and Chinese herbal medicine treatments, highlights the challenges that Chinese herbal medicine treatment for osteoporosis must overcome, and reveals the gap between emerging treatment methods and clinical applications, as well as potential directions for osteoporosis research, aiming to provide valuable references for future research on the prevention and treatment of osteoporosis.

## The fundamentals of bone and osteoporosis

The skeletal system is composed of 206 bones and is critical within the human body. First, the skeleton supports the shape of the body, forming the trunk and limbs and supporting movement; it also protects the brain, heart, lungs and other important organs, and some studies have shown that the skeleton has detoxification and endocrine functions [10–12]. Bones are very hard and metabolically active. Bone development begins as early as the beginning of pregnancy, and as the human body grows and develops, bone development continues until the age of 20 [13, 14]. Bone is composed mainly of periosteum, osseous tissue, endosteum and bone marrow. The outermost layer of every bone is the periosteum, which is a double-layered membrane. The outer fibrous layer is mainly responsible for supporting the rigid structure of bone. The inner cambium is rich in bone progenitor cells, which are important for the initial growth and development of bone and the repair of future damage. Osseous tissue is a mineralized tissue located between the periosteum and endosteum. Although osseous tissue does not contain bone marrow, the presence of mesenchymal stem cells in bone tissue is similar to that in bone marrow. Compared with the first two components, relatively few studies have investigated the endosteum, which originates from the periosteum. However, unlike the periosteum, the endosteum is very thin and plays an irreplaceable role in bone; it can not only promote bone repair but also control bone overgrowth and prevent the production of ectopic bone. Located in the deepest part of the bone, bone marrow is a key organ comprising hematopoietic stem cells and can be used for bone regeneration. At the cellular level, bone cells are composed of osteoblast lineages and osteoclast lineages. Osteoblasts are responsible for the continuous regeneration of bone and originate from mesenchymal stem cells. The main function of osteoclasts is the absorption of bone with aging, and osteoclasts are derived mainly from hematopoietic stem cells, such as macrophages and multinucleated giant cells. During the growth and development of the human body, bone formation and bone absorption are dynamic processes, and only a balance between the two can maintain the normal physiological structure and body function of the bone [15, 16]. If the balance between bone resorption and bone formation in the body is disrupted for various reasons, osteoclasts are activated, the absorption of bone significantly increases, the function of osteoblasts, i.e., new bone formation, is suppressed, bone mass decreases, bone metabolism regulation is disrupted, and bone density decreases, resulting in OP [17, 18].

## Molecular pathogenesis of OP

Understanding underlying molecular mechanisms is a prerequisite for disease treatment. This review introduces the molecular mechanisms of OP from four aspects: genetic factors, environmental factors, signaling pathways, and bone remodeling.

### Genetic factors

Genetic factors can affect bone mass and structure. Studies have shown that 50%−80% of the differences in bone mass among individuals are determined by genes. Using genome-wide association study technology, nearly 600 genes related to OP and fractures have been identified [19, 20]. Genetic factors include Deoxyribonucleic Acid/Ribonucleic Acid (DNA/RNA) methylation, histone modification, nucleosome localization, noncoding RNA, and chromatin configuration. DNA methylation can suppress important transcription factors, in turn reducing the expression of related genes and affecting the growth activity of many cells in bone. Recent research on DNA methylation and OP has focused mainly on changes in bone mineral density and risk factor markers of OP. However, relatively little research has investigated the mechanism of methylation in human osteoblasts at the whole-genome level, and therefore, more research is needed to provide new directions for the diagnosis and treatment of OP [21, 22]. Among noncoding RNAs(ncRNAs), microRNAs(miRNAs) regulate gene expression related to cell growth and apoptosis, and miRNAs are important for the maintenance of bone homeostasis. Serum miRNA expression increases significantly in OP fracture patients. miRNAs belong to the ncRNA family. Members of this family can recruit histone modifiers to regulate gene expression. On the one hand, miRNAs can degrade mRNA, and on the other hand, they can inhibit the translation of target genes, thus affecting the occurrence and development of osteoblasts and OP. Research has confirmed that when miR-218 activity is decreased, osteoblast activity is simultaneously inhibited, and bone mass further decreases. miR-188 and miR-218 are considered therapeutic targets for the treatment of OP [23, 24]. Another form of genetic influence is histone modification. In the presence of relevant factors, histones undergo posttranslational modification, which further affects gene transcription [25, 26]. In the field of bone metabolic diseases, histone acetylation and histone methylation are hotspots in research. Methyltransferases and demethylases play crucial roles in regulating gene expression in osteoblasts and osteoclasts [27, 28]. In-depth research on the genetic mechanisms can provide insight on the pathogenesis of OP and new ideas for the treatment of OP.

**Fig. 1:**
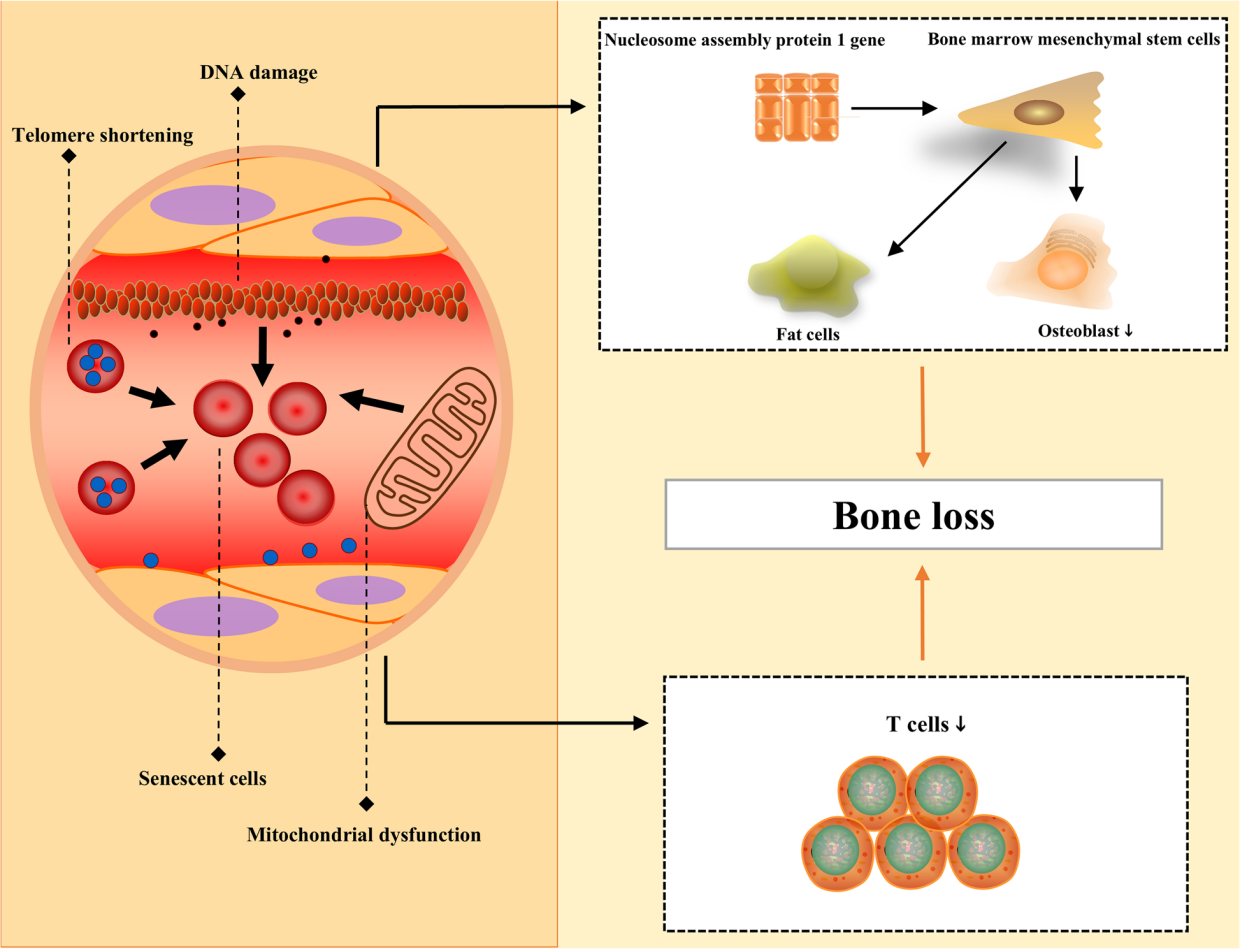
Aging of the body can cause OP. With increasing age, the number of senescent cells in the body gradually increases, and senile OP is a typical manifestation of senescence. Cell senescence is the result of many abnormal factors, including telomere shortening, DNA damage, and the physiological dysfunction of mitochondria. Under the influence of aging, the expression of the nucleosome assembly protein 1 gene is strongly affected, and the osteogenic differentiation ability of bone marrow mesenchymal stem cells is clearly suppressed. The aging of bone directly causes the abnormal transformation of bone marrow mesenchymal stem cells into fat cells, and T cells can also reduce the physiological function of cells during aging, resulting in bone loss. OP osteoporosis, DNA deoxyribonucleic acid

With increasing age, the number of senescent cells gradually increases and affects the body; for bones, senile OP is a typical manifestation of aging. Cellular senescence is the result of a variety of abnormal factors in the body, such as the wear and tear of telomeres, DNA damage, and the physiological dysfunction of mitochondria. At present, aging is a target of many scholars who are interested in the treatment of OP. Bone marrow mesenchymal stem cells (BMSCs) are important for bone regeneration. However, with increasing age, the osteogenic differentiation ability of BMSCs clearly decreases [29]. In addition, bone aging directly causes the abnormal transformation of bone marrow mesenchymal stem cells into fat cells, and over time, cells age, ultimately resulting in bone loss, promoting the progression of senile OP [30–32]. Nucleosome assembly protein 1 belongs to a relatively conserved gene family, plays a role in gene transcription, and participates in cell proliferation and expression. The expression of the nucleosomal assembly protein 1 gene is strongly affected during aging and is positively correlated with the weakening of the physiological capacity of bone marrow mesenchymal stem cells [33–35]. Senescent cells can be characterized by changes in original staining, development arrest, abnormal increases in metabolism, and abnormal apoptosis. In the body, senescent cells exhibit an aging-related secretion phenotype. Senescent cells may have the ability to evade immune cells, leading to their accumulation; however, the specific mechanism is still unclear [36, 37]. Senescent cells are clearly present in the microbial environment of bone and are present among a variety of cell types, such as bone cells and immune cells. Among them, the most studied cell population is the T cell population, whose physiological function decreases with aging; in contrast, relatively few studies have investigated senescence-related processes in B cells. The adverse effects of cellular senescence in bone are numerous; however, senescent cells may also have some physiological functions. The beneficial effects of senescent cells may involve damaged tissue repair. At the skin level, if injured, aging fibroblasts and endothelial cells immediately respond to and secrete the platelet-derived growth factor AA to promote rapid wound healing. Senescent cells can initiate rapid repair, possibly because they secrete specific cell and growth factors [38, 39]. In bone diseases, cellular senescence plays a very important role in senile OP, and therapies targeting cellular senescence are particularly important. Western medicine treatments, of which there are several options, include teriparatide and romozumab (Fig. 1).

### Environmental factors

OP results from the combined effects of genetic and environmental factors. Environmental factors include insufficient sunlight exposure, air pollution, dietary alterations, and unhealthy living habits, among others. Vitamin D plays an important role in the normal metabolism of bones. Ultraviolet B (UVB) light is an important environmental factor affecting the synthesis of vitamin D. Changes in season, latitude, and weather affect UVB exposure. Age, skin pigmentation, and duration of sun exposure are individual factors that influence vitamin D status (Wacker and Holick 2013). A cross-sectional study revealed that in the sun-deprived Sichuan Basin of China, the prevalence of vitamin D deficiency among adult women is very high in the winter; in sunny plateau areas, the 25(OH)D levels of young women with longer sun exposure are higher than those of their peers [40–42]. Air pollution has been identified as a risk factor affecting bone density, and its adverse effects on bones are particularly significant in industrialized areas [43]. Data indicate that people who are exposed to long-term air pollution have a 15% increased risk of developing OP. Long-term exposure to fine particulate matter (PM) can activate receptor activator of nuclear factor-κB ligand 1 (RANKL). Moreover, long-term exposure to polluted air can promote the release of inflammatory factors. Furthermore, a polluted air environment can reduce the amount of UVB that reaches the Earth's surface, inhibit the formation of vitamin D, reduce bone formation, and affect the bone remodeling process [44]. Studies have shown that air pollution induces oxidative stress in osteocytes and that polluted fine particulate matter inhibits wingless-related integration site (Wnt) signaling activity in bones, thereby suppressing the bone formation process and reducing bone mass [45, 46]. Moreover, exposure to fine particulate matter is positively correlated with the serum levels of bone resorption markers. Air pollution can affect both bone resorption and bone formation, inhibit bone remodeling, and cause bone loss [47]. Smoking and alcohol consumption can also promote the occurrence of OP. Smokers have lower trabecular bone volume and average trabecular thickness, with reduced bone formation [48]. The chemical components in tobacco smoke have been indicated to cause bone mineral loss in animal models. In human experiments, smoking has been shown to be positively correlated with bone mineral loss, and the risk of fractures in smoking patients is significantly increased. Additionally, Parathyroid Hormone (PTH) levels in smokers are significantly reduced but return to normal levels after quitting smoking. Therefore, smoking can affect both bone resorption and bone formation [49, 50]. Compared with that among abstainers, the risk of osteoporosis among people who drink 0.5–1 cup of alcohol per day is 1.38 times greater, the risk of osteoporosis among people who drink 1–2 cups per day is 1.34 times greater, and the risk of osteoporosis among people who drink two cups or more per day is 1.63 times greater [51].

### Signaling pathways

**Fig. 2:**
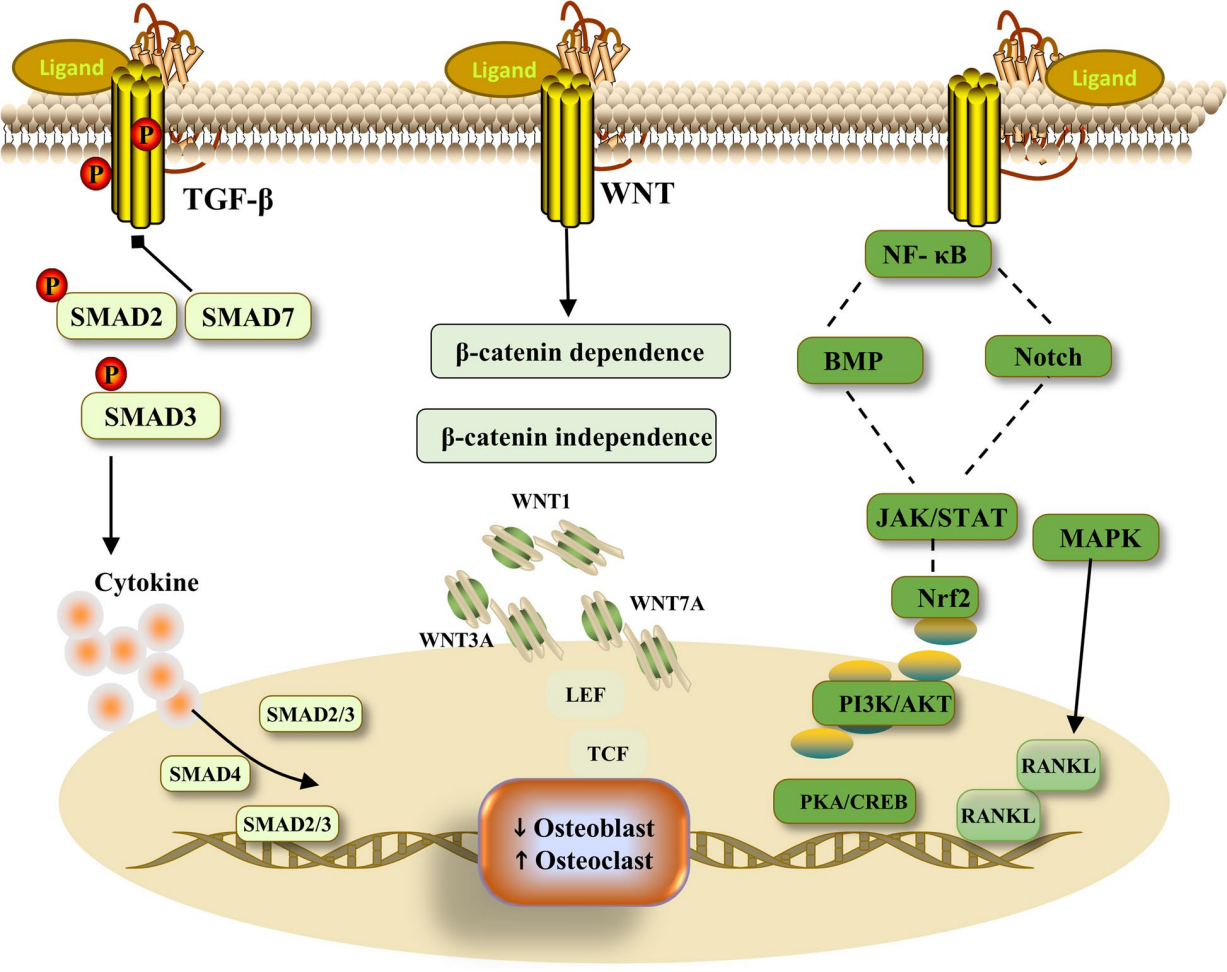
The abnormal activation of signaling pathways can affect bone metabolism. TGF-β can activate downstream SMAD signaling and interact with a variety of cytokines to affect bone metabolism. The Wnt signaling pathway can be divided into β-catenin-dependent and β-catenin-independent pathways. In the study of the human body, there are up to 19 Wnt ligands. If the activity of any Wnt signaling pathway in the body is impaired, normal bone metabolism is affected. The NF-κB and Notch signaling pathways inhibit osteoblast development and promote osteoclast differentiation. MAPK signaling mainly includes the ERK signaling, JNK signaling and p38 MAPK signaling pathways. Regulated by RANKL, MAPK promotes the differentiation of osteoclasts. In addition, other signaling pathways, such as the JAK/STAT, BMP and Nrf2 pathways, affect bone metabolism. TGF-β transforming growth factor-β, SMAD suppressor of mother against decapentaplegic, Wnt wingless-related integration site, NF-κB nuclear factor kappa-B, MAPK mitogen-activated protein kinase, ERK extracellular regulated protein kinases, JNK c-Jun n-terminal kinas, RANKL receptor activator of nuclear factor kappa-B ligand, JAK/STAT janus kinase/signal transducer and activator of transcription, BMP bone morphogenetic protein, Nrf2 nuclear factor erythroid 2-related factor 2,PI3K/AKT phosphoinositide 3-kinase/protein kinase B,PKA/CREB protein kinase A/cAMP response element binding protein,LEF lymphoid enhance factor,TCF t-cell factor

Normal bone metabolism is regulated by many signaling pathways, including the Transforming growth factor—β (TGF-β), Wnt, Nuclear factor kappa-B (NF-κB), and Mitogen-activated protein kinase (MAPK) pathways. Normal TGF-β signaling pathway activity plays an important role in maintaining skeletal system homeostasis. In bone, TGF-β originates from osteoblasts and is inactive after production; however, TGF-β is activated during bone resorption [52]. Many cells in the body can secrete TGF-β, including T lymphocytes, macrophages and platelets. TGF-β has three isoforms: TGF-β1, TGF-β2 and TGF-β3 [53]. TGF-β can activate the downstream Suppressor of Mother against Decapentaplegic (SMAD) signaling pathway, interact with a variety of cytokines, affect bone metabolism, inhibit TGF-β signaling overactivation, promote bone homeostasis, and prevent bone loss [54]. The Wnt signaling pathway is active in many cells throughout the body and is involved in many cellular functions; it can be specifically divided into two types: the classical pathway (β-catenin-dependent pathway) and the nonclassical pathway (β-catenin-independent pathway). The Wnt signaling pathway is very important for tissue embryo growth and development [55]. Wnt has a very large number of ligands, and in studies in humans, as many as 19 ligands have been identified. A variety of ligands also play regulatory roles in the skeletal system. For example, Wnt1 promotes the development of osteoblasts and inhibit the formation of osteoclasts; Wnt3A inhibits the apoptosis of undeveloped osteoblasts; and Wnt7A plays a regulatory role in the development of limbs and the skull [56]. If the Wnt signaling pathway is impaired, the normal metabolism of bone is affected. The classical Wnt signaling pathway has been extensively studied by scholars worldwide for the treatment of OP [57]. MAPK has many biological functions, including participating in inflammatory responses, promoting apoptosis, and participating in the activity of some genes. There are three main levels of MAPK signal transduction, namely, MAPK, Mitogen-Activated Protein Kinase Kinase (MAPKK), and Mitogen-Activated Protein Kinase Kinase Kinase (MAPKKK). In the skeletal system, MAPK promotes the proliferation of osteoclast precursors. Osteoclasts are regulated by many cytokines, among which macrophage colony-stimulating factor and RANKL are particularly important. Macrophage colony-stimulating factor and RANKL play roles in regulating MAPK signaling [58]. The MAPK family is very large and includes Extracellular Regulated Protein Kinases (ERK), c-Jun N-terminal Kinas (JNK)and p38 MAPK, and the ERK, JNK and p38 MAPK signaling pathways promote osteoclast differentiation [59]. The NF-κB signaling pathway, which plays important roles in inflammation and immune regulation, is involved in cell growth and development and apoptosis. The NF-κB signaling pathway is classified into classical and nonclassical pathways. The NF-κB signaling pathway has been extensively studied in cancer and autoimmune diseases and has been well studied in the skeletal system. In the process of osteoclast formation, NF-κB signaling pathway activation is a key step, and many inflammatory factors affect osteoclasts by activating the NF-κB signaling pathway. NF-κB signaling can inhibit osteogenic differentiation [60]. Notch is a very important signaling molecule that is directly involved in the physiological function of cells. Mutations in Notch are significantly associated with inherited bone disorders, such as brachydactyly. Notch signaling pathway dysregulation can lead to a variety of bone diseases. Notch inhibits the activity of the Wnt signaling pathway and hinders the development of osteoblasts. The structures of Notch1 and Notch2 are similar, but the isoforms have very different effects. Notch1 inhibits osteoclast generation, whereas Notch2 promotes osteoclast differentiation. In addition, Notch signaling activation can adversely affect the healing of fractures [61]. The Phosphoinositide 3-Kinase/Protein Kinase B (PI3K/AKT) signaling pathway plays important roles in cancer diseases; it regulates many life activities in the body and participates in cell generation, differentiation and apoptosis. PI3K/AKT is influenced by a variety of factors in the body and interacts with other signaling pathways that have been shown to be involved in bone metabolism. PI3K/AKT signaling pathway activation promotes the development of osteoblasts and inhibits the growth of osteoclasts [62]. Janus kinase (JAK) belongs to the protein tyrosine kinase family and acts on the transcriptional activator Signal Transducer and Activator of Transcription (STAT). The JAK/STAT signaling pathway plays important roles in the regulation of hematopoietic factors and the immune system and is involved in the occurrence of many immune system diseases. In the skeletal system, the JAK/STAT signaling pathway is involved not only in the normal bone metabolism but also in the growth and differentiation of bone cells. The JAK/STAT signaling pathway promotes osteoclast development through multiple pathways [63]. In addition, many signaling pathways, such as the Bone Morphogenetic Protein(BMP), Nuclear factor erythroid 2-related factor 2 (Nrf2), and Protein Kinase A/cAMP Response Element Binding Protein (PKA/CREB) pathways, are involved in the occurrence and development of OP (Fig. 2).

### Bone remodeling process

The bone remodeling process is important for maintaining bone integrity and can be divided into four stages, namely, activation, resorption, reversal, and formation, that maintain bone stability. Under normal circumstances, osteoblasts and osteoclasts maintain a dynamic balance, thereby sustaining coordinated bone resorption and bone formation. In the OP state, bone resorption increases, bone formation decreases, the balance between osteoblasts and osteoclasts is disrupted, and bone mineral density decreases. Many factors affect the bone remodeling process, and complex molecular mechanisms interact with each other.

#### Oxidative stress

**Fig. 3:**
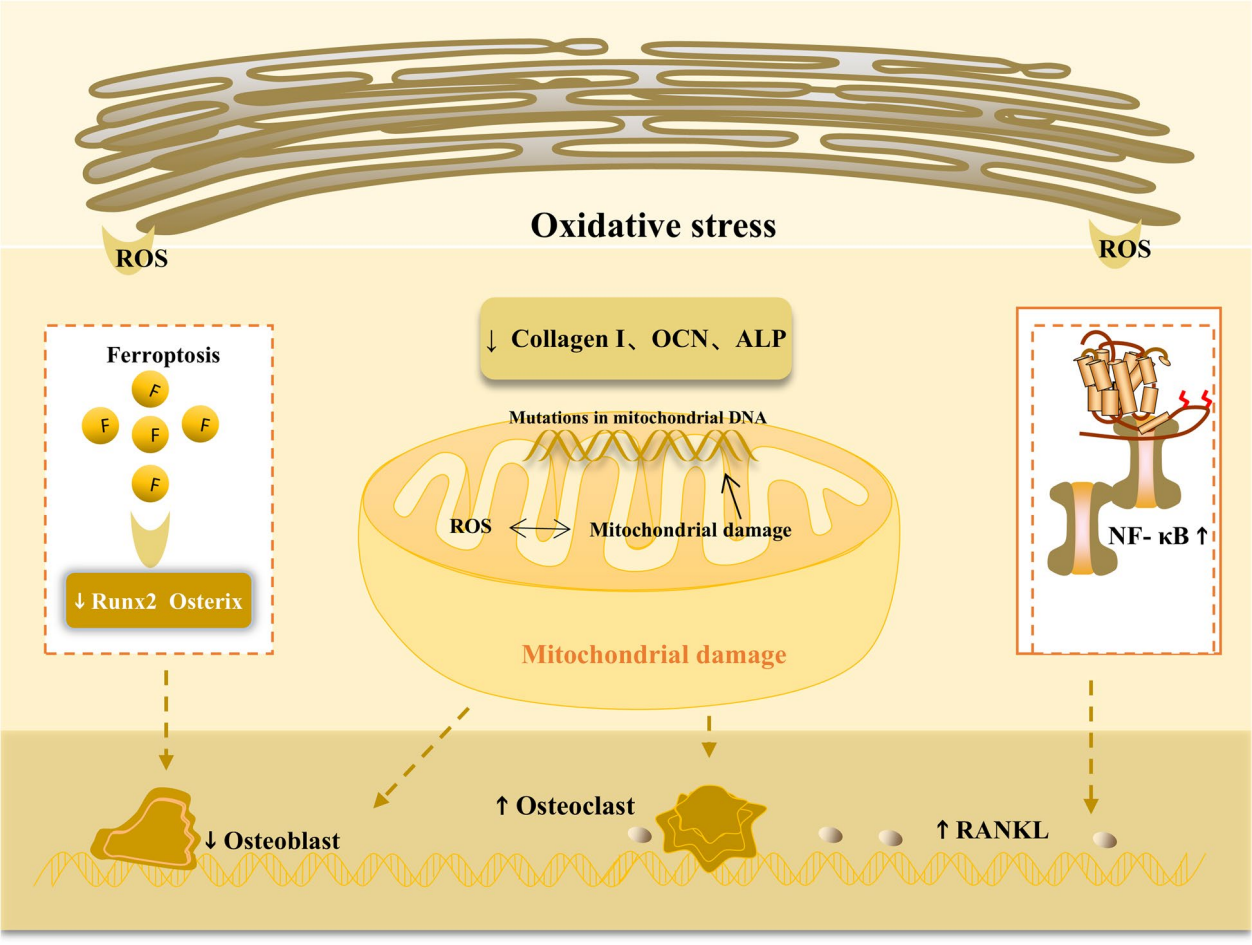
Oxidative stress plays an important role in regulating OP. Under oxidative stress, ROS increase abnormally and can significantly reduce the activity of Runx2 and osterix and affect the development of osteoblasts. Abnormal ROS accumulation can induce ferroptosis, further affecting osteoblasts. ROS can damage the normal function of mitochondria, and the loss of normal mitochondrial function affects neighboring mitochondria, leading to the release of a large number of ROS and inducing OP. Mutations in mitochondrial DNA are also associated with abnormal bone metabolism. Additionally, under oxidative stress, the activity of many antioxidant enzymes, such as collagen I, OCN and ALP, decreases, and osteogenic differentiation is affected. NF-κB signaling and RANKL activity are increased, and osteoclast differentiation is increased. OP osteoporosis, Runx2 runx family transcription factor 2, ROS reactive oxygen species, OCN osteocalcin, ALP alkaline phosphatase, NF-κB nuclear factor kappa-B, RANKL receptor activator of nuclear factor kappa-B ligand

Oxidative stress plays an irreplaceable role in normal activities and damage repair in body tissues. Oxidative stress refers to a disruption in the balance of oxidation and antioxidant functions in the body; most of the body is in an oxidative state. Oxidative stress plays a key role in regulating OP. Oxidative stress can be caused by many factors, including physiological conditions, such as natural aging and metabolic and hormonal changes with age, and pathological conditions, such as inflammatory states and the use of certain medications [64, 65]. The main mechanism of oxidative stress is the abnormal increase in enzymes that produce reactive oxygen species (ROS) and the significant inhibition of the activity of various antioxidant enzymes in the body [66]. ROS are free radicals in the body that have been shown to disrupt normal bone metabolism in a variety of ways. First, ROS can significantly reduce the activity of Runx family transcription factor 2 (Runx2) and Osterix and further affect the development of osteoblasts. Furthermore, ROS can promote osteoclast differentiation and bone loss. In addition, the activity of many antioxidant enzymes is inhibited. Various antioxidant enzymes, such as glutathione, increase the activity of collagen I, Osteocalcin (OCN) and Alkaline phosphatase (ALP) in the body and promote osteogenic differentiation. In addition, antioxidant enzymes can inhibit the activation of the NF-κB signaling pathway, thereby suppressing RANKL activity and ultimately inhibiting osteoclast formation. The abnormal accumulation of ROS can affect iron metabolism, induce ferroptosis, and inhibit normal Wnt signaling, further affecting the development of osteoblasts, disrupting bone homeostasis, and causing OP [67]. Mitochondria not only provide energy support but also participate in many metabolic activities in the body. In addition to red blood cells, all cells have mitochondria, which are important for maintaining the normal physiological function of cells. Mitochondria produce ROS through processes involving the Electron Transport Chain (ETC)and Nicotinamide Adenine Dinucleotide Phosphate (NADPH) oxidase; however, ROS damage the normal function of mitochondria, and the loss of normal mitochondrial function affects neighboring mitochondria, releasing many ROS and ultimately inducing OP [68]. Mitochondria are dependent on nuclear factor E2 P45-related factor 2 for the regulation of excess ROS; mice lacking nuclear factor E2 P45-related factor 2 have significantly reduced bone mass [69]. Mutations in mitochondrial DNA are significantly associated with a variety of inherited bone diseases. Excess iron in the body affects the physiological structure of mitochondria, further stimulating the production of ROS and cytokines, thus driving apoptosis. The use of antioxidant inhibitors can reverse these pathological changes [70] (Fig. 3).

#### The immune system and inflammation

**Fig. 4:**
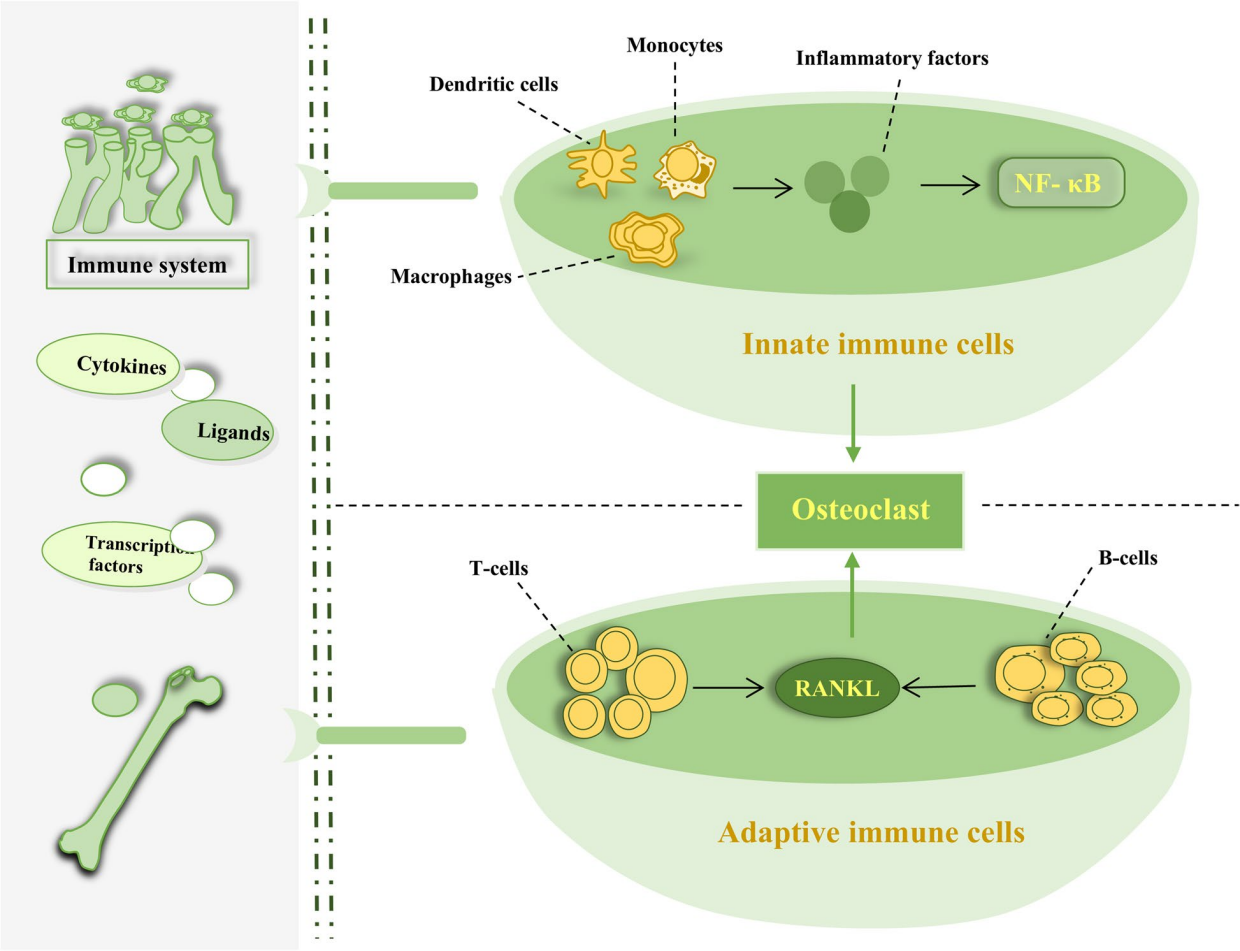
There is a strong link among the immune system, inflammation and OP. The skeletal system and the immune system have the same cytokines, transcription factors and ligands. Immune cells can be divided into innate and adaptive types. Innate immune cells include dendritic cells, monocytes, and macrophages; these cells promote the progression of inflammation, thereby increasing the function of osteoclasts and promoting bone loss. Representative inflammatory factors, including IL-6 and TNF-α, activate the NF-κB signaling pathway, causing inflammatory factor responses downstream of the signaling pathway and further stimulating the function of osteoclasts. Adaptive immune cells include T lymphocytes and B lymphocytes. In the absence of stimulation, T lymphocytes inhibit osteoclast function; if T lymphocytes are activated by RANKL, they play a role in strengthening osteoclasts. B cells can produce RANKL, which directly promotes bone loss. OP osteoporosis, NF-κB nuclear factor kappa-B, RANKL receptor activator of nuclear factor kappa-B ligand, IL-6 interleukin-6, TNF-α tumor necrosis factor-alpha

There is a complex relationship among the immune system, inflammation and OP. At present, many Western medicine drugs for the treatment of OP play a therapeutic role by regulating immune and inflammatory states. Many years ago, the term “osteoimmunology” appeared in the journal Nature, and RANKL was discovered [71, 72]. The immune system is very complex and important for organisms. In nature, in both plants and animals, the immune system is needed to maintain normal physiological function. Both the skeletal system and the immune system are closely related and share factors such as cytokines, transcription factors and ligands. The skeletal system and immune system work together to maintain the normal physiological functions of bone, such as the hematopoietic function of the bone marrow and the normal metabolic function of minerals [73–76]. Rheumatoid arthritis is a disease characterized by interactions between the skeletal system and the immune system; it is a bone disease induced by abnormal immune activity. In addition, periodontitis, ankylosing spondylitis and other diseases are important driving forces that promote the development of bone immunology [77–79]. RANKL is an important factor that connects the skeletal system and the immune system; it directly participates in the function of the body's immune system. Bone remodeling requires the joint action of osteoblasts, osteoclasts and other cells in the bone, and therefore, there needs to be a communication hub between these cells: RANKL is an important factor in this hub [80, 81]. Inflammation can promote the development of OP, and for people with Crohn's disease, rheumatoid arthritis and other diseases, the probability of OP is significantly increased [82–84]. Inflammation plays a role in the body in various ways, including through a variety of cytokines. Inflammation can first result in the production of many soluble cytokines, which further stimulate immune cell responses. Various cytokines, including Interleukin-6 (IL-6) and Tumor Necrosis Factor-alpha (TNF-α), can initiate the NF-κB signaling pathway and ultimately cause the response of downstream inflammatory factors, resulting in inflammation. This process stimulates and promotes the function of osteoclasts and affects the physiological function of bones and normal metabolism [85–88]. Immune cells can be divided into innate and adaptive cells. Innate immune cells include dendritic cells, monocytes, macrophages, innate lymphocytes, mast cells, and natural killer cells. Dendritic cells promote the progression of inflammation, thereby increasing the function of osteoclasts and promoting bone loss. Both monocytes and macrophages are relatively common immune cells that participate in the inflammatory response. As another branch of the immune system, innate lymphocytes play a role in preventing the invasion of pathogenic microorganisms and maintaining the physiological function of immune tissue. Mast cells are very important cells in the immune system; they are also found in the skeletal system and are associated mainly with allergic reactions. Natural killer cells can increase the activity of osteoclasts, inhibit the physiological function of osteoblasts, and thus affect the normal metabolism of bones. Adaptive immune cells include T lymphocytes and B lymphocytes. T lymphocytes play two roles in maintaining normal bone metabolism: T lymphocytes can inhibit the function of osteoclasts without any stimulation, and if T lymphocytes are activated by RANKL, they play a role in activating osteoclasts. B cells can produce RANKL, which directly promotes bone loss, and can also produce osteoblast inhibitors, which inhibit the physiological function of osteoblasts by activating various signaling pathways [89–93]. Immune cells and inflammatory cells play important roles in the normal physiological activities of bone (Fig. 4).

#### Iron metabolism

Iron is essential for maintaining normal physiological activities in the body; it is involved in cell growth and development and is also a key component of the oxidative stress system. The normal metabolism of iron in the body is complex and balanced and includes the production of iron, the utilization of iron by cells and tissues, the storage of surplus iron and the consumption of iron. Iron, which the body takes in through the diet, is first absorbed by the intestinal epithelium and then participates in various cycles for use by the body or is stored in ferritin. The iron transporter-sideromodulin regulatory axis regulates iron in the body to maintain an iron balance. If iron accumulates abnormally in the body, sideromodulin is activated, preventing iron from entering the circulatory systems in various ways [94, 95]. Iron can promote the production of free radicals, and ferrous iron can generate hydroxyl radicals, which are ROS. If the body is overloaded with iron and abnormal ROS accumulation occurs because of a variety of factors, the oxidative stress response is activated, resulting in the disruption of cytokines and many biological systems [96]. Abnormal iron metabolism, such as an abnormal increase in iron production or an abnormal decrease in iron excretion, can trigger oxidative stress and cause ferroptosis. Ferroptosis plays a regulatory role in a variety of diseases, such as malignant tumors, liver diseases, and cardiovascular diseases. Ferroptosis is associated with OP at the same time [97, 98]. First, excessive iron accumulation results in excess ROS production, activates oxidative stress, affects a variety of normal signaling pathways, and inhibits the function of osteoblasts; ferroptosis also significantly suppresses the function of bone marrow mesenchymal stem cells and further suppresses the formation of osteoblasts. Scholars have conducted in vitro experiments to assess the effects of ferroptosis on the mouse calvarial preosteoblastic cell line, subclone-E1(MC3T3-E1), and the results revealed that the osteogenic differentiation ability of MC3T3 cells under conditions of ferroptosis significantly decreased. In addition, iron metabolism affects osteoclasts. Iron accumulation leads to excessive ROS accumulation, which causes the abnormal activation of the MAPK and NF-κB signaling pathways, promotes the function of osteoclasts, and causes bone loss and OP [99, 100]. Iron metabolism is also involved in the ROS and oxidative stress systems. The connections among various systems, cells, and tissues in the body are complex and mysterious. Oxidative stress occurs mainly when the balance between oxidation and antioxidation is disrupted. Many inducing factors, including physiological and pathological factors, ultimately lead to an abnormal increase in ROS levels. An abnormal increase in ROS levels subsequently affects iron metabolism. In the case of abnormal iron metabolism, which is due mainly to iron overload, excessive ROS are produced, activating oxidative stress. Iron metabolism, oxidative stress, and ROS are closely connected. However, the role of ROS in iron metabolism and oxidative stress differs.

#### Gut microorganisms

The gastrointestinal tract has a large number and variety of microorganisms, and the intestinal microbiota has a symbiotic relationship with cells to maintain many physiological functions within the body. The gut microbiome includes bacteria, viruses, and fungi, which coexist harmoniously in the gut. The dysregulation of gut microbes is associated with a variety of diseases, such as diarrhea, obesity, and metabolic diseases. Once the gut microbiota is established, it remains relatively stable. As the body grows and develops, several factors affect the composition of gut microbes, such as diet, drug use, and inflammation [101–103]. The intestinal microbiota not only affects the environment and physiological functions of the adjacent intestine but also extensively affects the physiological activities of bones, the liver, the brain and other organs. Inflammatory bowel disease, metabolic diseases, arthritis, rheumatoid arthritis, OP and other diseases are related to intestinal flora disorders [104, 105]. The gut microbiota plays important roles in bone loss and bone metabolism. Gut microbes can affect the skeletal system in a variety of ways. The skeletal system requires various hormones, such as parathyroid hormone, estrogen, and gastrointestinal hormones, to maintain its basic physiological activities. The production of these hormones is regulated by the intestinal microbiota, and metabolic disorders involving each hormone affect normal bone metabolism. For example, estrogen deficiency can cause an imbalance in the intestinal flora, which subsequently affects the skeletal system and causes bone loss, and estrogen supplementation can have a favorable effect by restoring the normal environment of the intestinal flora and inhibiting bone loss [106, 107]. An imbalance in intestinal microbes leads to increased intestinal permeability and damage to intestinal compaction-linked proteins, which further causes bacterial displacement and the spread of inflammation. In intestinal models, inflammation is significantly positively correlated with bone loss. Some bacteria among the intestinal microbiota can stimulate T helper 17 cell (Th17 cell) production, which can stimulate inflammatory cell Interleukin 17 (IL-17) secretion and RANKL production, further increasing osteoclast activity and bone absorption [108]. Short-chain fatty acids, mainly acetate, propionate and butyrate, are metabolites of intestinal microbial fermentation of dietary fiber. Short-chain fatty acids play important roles in maintaining normal intestinal permeability and the intestinal barrier, which can reduce the production of inflammatory factors and prevent bone loss. In addition, short-chain fatty acids directly affect osteoblasts, regulate osteoblast formation, promote bone formation, and inhibit osteoclast function. Short-chain fatty acids also promote the differentiation of bone progenitor cells by regulating the function of bone marrow stromal cells, further regulating bone metabolism, and maintaining normal bone density and bone health [106, 109].

#### Autophagy

Autophagy is a self-degrading process within cells, and its process is very conserved. Autophagy plays an important role in the maintenance of cell function and is directly involved in the development of cells and the maintenance of their renewal ability. There are three forms of autophagy, namely, macroautophagy, chaperone-mediated autophagy and microautophagy. Autophagy allows cells to continue growing even when there is a nutrient deficiency. Autophagy can regulate immunity and play a regulatory role in a variety of diseases, such as neurological diseases, cardiovascular diseases and musculoskeletal diseases [110]. Osteoblasts and osteoclasts maintain a normal autophagic state to maintain viability and balance bone metabolism. The disruption of the autophagic balance affects bone metabolism and causes OP. Reduced autophagy in osteoblasts reduces their lifespan. Osteocytes are terminally differentiated cells of the osteoblast lineage. Abnormal autophagy can lead to abnormal osteocyte function. Autophagy is involved in the regulation of bone metabolism by various growth hormones. Mitophagy can inhibit oxidative stress, regulate the functions of osteoblasts and osteoclasts, and maintain normal skeletal physiological activities [111]. In addition, multiple signaling pathways disrupt the differentiation balance between osteoblasts and osteoclasts by influencing the level of autophagy [112].

#### Other factors

Many other factors may affect the bone remodeling process, such as postmenopausal body status, excessive smoking, excessive alcohol consumption, glucocorticoid use, and the use of certain medications. The main cause of OP in postmenopausal women is estrogen deficiency. Glucocorticoids can increase the production of Peroxisome Proliferator-Activated Receptor Gamma 2 (PPAR-2), resulting in limited osteoblast growth, reduced bone formation, and bone loss. Compared with a control group, an experimental group receiving glucocorticoid treatment had significantly lower bone density. In addition to glucocorticoids, drugs such as proton pump inhibitors, chemotherapeutic agents, and antidepressants may affect bone remodeling, cause osteopenia, and induce OP [113].

In summary, OP is the result of the combined effects of environmental and genetic factors; its molecular mechanisms involve signaling pathways, oxidative stress, inflammation, intestinal microbiota, genes, autophagy, and other processes. The influencing factors are complex and can interact with each other, leading to increased bone resorption, decreased bone formation, imbalanced bone remodeling, and reduced bone mass.

## Diagnosis of osteoporosis

The diagnosis of osteoporosis requires a combination of medical history, symptoms, and auxiliary examination results. Medical history includes personal history, family history, medication history, etc., for example, age, sex, medication use, and genetic disease history. Symptoms include bone pain, fractures, and limited mobility. Auxiliary examination results include laboratory tests and imaging examinations. Laboratory tests include routine blood tests, liver and kidney function tests, and electrolyte, alkaline phosphatase, 25-hydroxyvitamin D3, parathyroid hormone, and routine urine tests. Imaging examinations include X-ray, Computed Tomography (CT), Magnetic Resonance Imaging (MRI), nuclear medicine, and bone mineral density (BMD) [114]. X-ray and CT are common imaging methods for diagnosing OP. X-rays are more widely used. MRI can avoid ionizing radiation, but standards for the diagnosis of OP are lacking. Nuclear medicine examinations are highly important for the diagnosis of secondary OP. BMD measurement methods include dual-energy X-ray absorptiometry (DXA), quantitative computed tomography, and peripheral DXA. Among them, DXA is the most widely used. A Chinese expert consensus indicates that OP can be diagnosed when BMD measurements and clinical symptoms suggest OP; however, if imaging examinations indicate an insufficiency fracture, osteoporosis can be diagnosed [115]. The diagnostic criteria for osteoporosis established by the World Health Organizatio (WHO), which are based on DXA, are mainly for postmenopausal women, women who have undergone oophorectomy, and men over 50 years old [114].

In addition, the DXA trabecular bone score (TBS), spectral CT, dual-energy CT, MRI measurements of fat, and AI technology have provided new options for the diagnosis of OP. The above technologies have improved the ability to predict fracture risk. However, owing to equipment access and cost, more extensive research should be conducted on diagnostic applications for OP [116–119].

## Therapeutic interventions for osteoporosis

The treatment of OP is extensive and can be divided into Western medicine treatment, Chinese herbal medicine treatment, nonpharmacological management and emerging therapeutic strategies.

### Therapeutic interventions involving Western medicine

There is no unified standard for the drug treatment of OP, thus requiring individualization in clinical practice. Drugs for treating OP can be divided into four main categories: antiresorptive drugs, anabolic drugs, dual-action drugs and other mechanism-based drugs. Currently approved drugs include bisphosphonates (alendronate, ibandronate, and zoledronic acid), parathyroid hormone analogs (teriparatide and abaloparatide), estrogen-related therapy (raloxifene-conjugated estrogens/bazedoxifene), RANK-ligand inhibitors (denosumab), sclerostin inhibitors (romosozumab), and calcitonin salmon [120]. Among them, parathyroid hormone analogs are anabolic drugs, sclerostin inhibitors are dual-action drugs, other drugs include antiresorptive drugs, and drugs with other mechanisms include active vitamin D and its analogs [121].

Bisphosphonates are traditional drugs for the treatment of OP. They can bind to bone minerals, inhibit the function of osteoclasts, and thus increase bone mass. Bisphosphonates are available in various formulations, including oral tablets, effervescent tablets, intravenous preparations, etc. They are inexpensive and are very commonly used as first-line drugs for the clinical treatment of OP. However, bisphosphonates cause adverse reactions, such as acute-phase injection reactions and gastrointestinal discomfort, and there is also a risk of osteonecrosis of the jaw. Denosumab is a biological agent and a human monoclonal antibody; it can inhibit RANKL activity and further suppress the formation of osteoclasts [122]. Raloxifene is an estrogen agonist that can reduce bone resorption and increase bone mineral density. Women with conditions such as venous thromboembolism, who are breastfeeding, and who are pregnant should avoid using raloxifene [123]. Calcitonin is a synthetic polypeptide hormone that can inhibit the function of osteoclasts; currently, it is not a first-line treatment for osteoporosis [124]. Relatively speaking, anabolic drugs and dual-action drugs are emerging as OP treatment options. Anabolic drugs include teriparatide and abaloparatide, both of which are parathyroid hormone analogs; they can stimulate osteoblasts, simultaneously stimulating Insulin-like Growth Factor-I (IGF-I)synthesis and inhibiting sclerostin, thus promoting bone formation [125]. Teriparatide was first applied in clinical practice and was the first approved osteogenic agent [126]. Abaloparatide was approved for clinical use by the U.S. Food and Drug Administration (FDA) in 2017; it binds to the same receptor as teriparatide does [127]. Dual-action drugs can promote bone synthesis while inhibiting bone resorption. Sclerostin is a glycoprotein secreted by osteocytes that inhibits Wnt signaling, leading to weakened osteoblast function. In addition, sclerostin enhances the activity of RANKL, resulting in increased osteoclast activity [128]. Currently, there are three anti-sclerostin monoclonal antibodies: blosozumab, setrusumab, and romosozumab. All of them have achieved good results in clinical trials. Romosozumab has been put into clinical use [129]. Research has shown that, compared with bisphosphonates, teriparatide and romosozumab lead to faster and greater increases in BMD, along with a substantial reduction in fracture risk [130, 131]. In terms of cardiovascular safety, real-world evidence has shown that both abaloparatide and teriparatide are safe [132]. However, compared with alendronate, romosozumab increases the risk of adverse cardiovascular events [133]. The European Medicines Agency has proposed that women who have had a myocardial infarction or stroke should avoid using romosozumab [134].

At present, there are many Western medicines for the treatment of OP, with a wide range of choices. Each type of drug has its own advantages and disadvantages. Antiresorptive drugs remain the main medications for the treatment of OP, but poor compliance and efficacy affect their application in clinical practice. Many emerging targeted drugs are being developed, such as cathepsin K inhibitors, Dickkopf WNT Signaling Pathway Inhibitor 1 (DKK1) inhibitors, which may provide new options for the treatment of OP [135]. In terms of clinical choices, various drugs are affected by factors such as price, contraindications, and adverse reactions, and therefore, treatment decisions should be made according to specific circumstances (Table 1).

**Table 1:** Summary of Western medicines for altering osteoporosis-related mechanisms

Drug name	Target	Development stage	References
Alendronate sodium	FPPS	Marketed	[136]
Ibandronate sodium	FPPS	Marketed	[137]
Pamidronate sodium	FPPS	Marketed	[138]
Risedronate sodium	FPPS	Marketed	[130]
Zoledronic acid	FPPS	Marketed	[139]
Minodronic acid	FPPS	Marketed	[140]
Raloxifene	ER	Marketed	[141]
Denosumab	RANKL	Marketed	[142]
Teriparatide	PTHrP	Marketed	[143, 144]
Abaloparatide	PTHrP	Marketed	[145]
Salmon Calcitonin	CGRP	Marketed	[146]
Eel Calcitonin	CGRP	Marketed	[147]
Ergocalciferol	VDR	Marketed	[148]
Cholecalciferol	VDR	Marketed	[149]
Calcifediol	VDR	Marketed	[150]
Calcitriol	VDR	Marketed	[151]
Alfacalcidol	VDR	Marketed	[152]
Eldecalcitol	VDR	Marketed	[153]
Romosozumab	SOST	Marketed	[154]
JMT103	RANKL	Ph2(NCT05278338)	*
Setrusumab	SOST	Ph3(NCT05125809)	*
Blosozumab	SOST	Ph2(NCT02109042)	*
AMG 167 SHR-1222	SOST SOST	Ph1(NCT01101048) Ph1(NCT04435158)	* *
Senolytics (Dasatinib + Querceti)	SASP	Ph2(NCT04313634)	*

^*^:https://clinicaltrials.gov/**.** FPPS farnesyl pyrophosphate synthase, CGRP calcitonin gene-related peptide, PTHrP parathyroid hormone-related protein, ER estrogen receptor, RANKL receptor activator of nuclear factor-κB ligand, VDR vitamin d receptor, SOST sclerostin, SASP senescence-associated secretory phenotype, Ph phase

### Therapeutic interventions with traditional Chinese medicine

Total flavonoids of Epimedium (EF) is among the active ingredients of Epimedium. EF Capsule, a Chinese patent medicine containing EF as the main active ingredient, has been approved for clinical use by the National Medical Products Administration of the People's Republic of China [155]. In addition, many other Chinese herbal medicines for the treatment of OP have emerged. Andrographis paniculata is a common Chinese herbal medicine with extensive clinical effects. Andrographolide is a diterpene lactone extracted from Andrographis paniculata; it can regulate immunity and inhibit inflammation. Results have shown that andrographolide promotes osteoblast differentiation, suppresses osteoclasts, inhibits apoptosis and delays senescence in rats. Moreover, results have shown that andrographolide has a short biological half-life and poor solubility, which may limit its clinical application in OP [156]. Pharmacological studies of Western medicine have shown that Dendrobium officinale inhibits tumor progression, delays aging and treats OP [157]. In a male aging mouse model, extracted antler bone calcium and bioactive peptides from antler bone were administered, and the effects on bone metabolism were assessed. The results showed that antler bone reduces age-related bone loss in mice and improves bone structure in aging mice [158]. Resveratrol is an edible polyphenolic plant antitoxin that has antioxidant and antiaging effects. Bone marrow mesenchymal stem cells from aged mice were cultured with different concentrations of resveratrol, and aging-related genes, osteogenic genes, signaling pathway-related genes, and ROS were assessed; the results revealed that resveratrol had antioxidant effects, activated AMPK signaling, decreased aging-related gene expression, and promoted osteogenic differentiation [159]. According to Western medicine pharmacology studies, the main active ingredients of Cnidii fructus include osthole, bergapten, and imperatorin and can alleviate inflammation, regulate the Wnt/β-catenin signaling pathway, and treat OP [160]. Emodin is among the main active components of rhubarb. In a rat model of OP induced by inflammatory bowel disease, emodin (30 mg/kg) was administered as an intervention, followed by assessments of inflammatory factors and bone metabolism indices. The results showed that emodin inhibits osteoclast differentiation and decreases the expression of tumor necrosis factor, macrophage-related factors and other inflammatory factors and has a good effect against OP [161]. Animal experiments revealed that salted E. ulmoides bark granules reduce the levels of interleukin-6 and tumor necrosis factor, alleviate the inflammatory state and protect bone structure after ovariectomy (OVX)-induced OP in rats [162]. Pharmacological studies have shown that galangal can inhibit the activation and activity of p38-MAPK and NF-κB signaling, thereby playing an anti-inflammatory role and alleviating OP [163]. Ginsenosides are among the main active components of ginseng. The ginsenoside Rb1 was administered to a dexamethasone (DEX)-induced OP rat model, and the expression of osteogenic differentiation-related genes and NF-κB pathway-related genes was evaluated in DEX-induced OP rat osteoblasts. The results revealed that Ginsenoside Rb1 (GRb1) inhibited the activation of NF-κB signaling and alleviated DEX-induced inflammation and OP in rats [164]. Cornus officinis contains many monomeric components, including flavonoids, tannins, and iridoids, and has a wide range of effects. Pharmacology studies have shown that Cornus officinis can control inflammation, regulate immunity, improve bone homeostasis, and delay the progression of OP [165]. Curcumin is the main active component of turmeric and can alleviate inflammation, inhibit bacterial growth, regulate immunity and lower blood sugar levels. In a previous study, osteoblast-like MG-63 human osteosarcoma cells were used to study the antiosteoporosis activity of a complex of gold nanoparticles and curcumin. The results revealed that the complex inhibits RANKL activity in bone marrow-derived macrophages and reduces osteoclast production [166]. Icariin is among the main components of icariin. Icariin can alleviate inflammation and OP. In vivo and in vitro experiments with mice and EI cells revealed that icariin weakens the activation of the NF-κB signaling pathway, inhibits the secretion of RANKL, increases the level of TGF-β1, and subsequently promotes the development of osteoblasts. Other studies have shown that icariin regulates the JNK/c-Jun signaling pathway and normal bone metabolism [167, 168]. Studies have shown that quercetin regulates signaling pathways such as Wnt/β-catenin and ERK/JNK. In addition, quercetin has antioxidant effects, ultimately promoting the development of osteoblasts, inhibiting osteoclasts, maintaining the physiological activities of bones, and alleviating OP. In addition, in MC3T3-E1 cells, treatment with quercetin activates the Nuclear Factor Erythroid 2-Related Factor 2/Heme Oxygenase-1 (Nrf2/HO-1) pathway, plays an antioxidant role, and thus plays a role in bone protection [169, 170]. Emodin is the main active component of rhubarb and can alleviate inflammation, regulate immunity, and regulate lipid metabolism, among other effects. In mouse MC3T3-E1 cells, the effect of emodin on bone metabolism was evaluated after the administration of 5 mg of emodin. The results showed that emodin activates the activity of PI3K-Akt/MAP kinase, thereby promoting osteoblast development and bone metabolism [161]. Matrine is among the main active components of Sophora flavescens and inhibits tumors, alleviates fibrosis and regulates immunity. In bone marrow monocytes (BMMs) and C57BL/6 mice, treatment with matrine inhibits the activity of RANKL and macrophage colony-stimulating factor and the abnormal activation of NF-κB, AKT and other signaling pathways, adversely affecting osteoclast generation and alleviating OP [171]. Polydatin is one of the extracts of Polygonum polygonum. In an OP model constructed from mouse preosteoblasts, polydatin inhibits the activity of the MAPK signaling pathway and alleviates OP [172]. Artesunate is the main active ingredient of artemisinin. In an in vitro study of lipopolysaccharide-stimulated osteoclasts from mice, artesunate significantly decreased the expression of Toll-like Receptor 4/Tumor Necrosis Factor Receptor-Associated Factor 6 (TLR4/TRAF6)and downstream Phospholipase C gamma 1-Calcium ion (PLCγ1-Ca) and further inhibited osteoclast generation [173]. Pharmacological studies have shown that Ginkgo can inhibit the activation of NADPH oxidase, promote the activation of AMPK signaling, and regulate Signal Transducer and Activator of Transcription 3/Janus Kinase 2 (Stat3/JAK2), Nrf-2, mammalian target of rapamycin (mTOR), BMP and other signaling pathways, thus alleviating OP [174]. Morinda officinalis polysaccharide is the key active component of polysaccharides in Morinda officinalis and can delay aging and regulate blood sugar levels. Morinda officinalis polysaccharides have significant antioxidant effects and are commonly used to treat OP [175]. Crocin is the main active ingredient of saffron. In rats, 5–10 mg/kg crocin was administered per day for 12 weeks, and the levels of inflammatory factors such as TNF-α and IL-6 and oxidative stress factors in the epiphyseal tissues of the rats significantly decreased. In addition, crocin regulates mitochondrial function to play an antioxidant role, ultimately conferring bone protection [176].Cordyceps militaris is one of the best sources of cordycepin. As an in vitro model, BMMSCs were treated with increasing concentrations of Hydrogen Peroxide (H2O2) for 24 h to induce oxidative damage, and OVX and aged mice were used as in vivo models; the in vitro and in vivo models were treated with Cordycepin. The results revealed that Cordycepin reduced the level of oxidative stress in both the mouse and cell models, promoting the development of osteoblasts, inhibiting the activity of osteoclasts and preventing bone loss [177]. Gastrodin is the main active ingredient of Gastrodia elata, and pharmacological studies have shown that Gastrodin can improve circulation, increase anti-inflammatory activity, and control blood pressure. Iron dextran-induced iron overload in mice was used as an in vivo model, and glutamate-treated hippocampal neurons (HT-22) were used an in vitro model of ferroptosis. The models were treated with the drug Gastrodin, and the results showed that Gastrodin alleviated nerve damage in ferroptosis models by modulating NRF2/HO-1 signaling; Gastrodin also inhibited ferroptosis and protected osteoblasts through antioxidant effects [178]. Curculigoside is the active ingredient of Curculigo orchioides Gaertn. In vivo and in vitro experiments using mice and MC3T3-E1 cells revealed that curculigoside reduces the production of ROS, inhibits Insulin-like Growth Factor Recepto/Protein Kinase B (IGFR/AKT) signaling pathway activation, and regulated the expression of the Forkhead Box O1 (FoxO1) target gene Manganese Superoxide Dismutase (MnSOD), thereby inhibiting the apoptosis of iron overload cells and protecting the normal metabolism of bone [179]. QinE pill (QEP) is a commonly used Chinese medicine compound prescription in clinical practice. In rats with OVX-induced postmenopausal osteoporosis, QEP reduced ferroptosis and increased the activation of the Protein Kinase B/Phosphoinositide 3-Kinase (AKT/PI3K) pathway to promote osteoblast development and prevent bone loss; thus, QEP is a potential drug for treating OP [180]. Resveratrol is among the most studied natural polyphenols. Studies have shown that resveratrol has a strong prebiotic effect and can regulate the intestinal flora and maintain the balance of the intestinal bone axis [181]. Ginseng is a very common herbal medicine, and ginsenoside is among its main active ingredients. Mice with intestinal flora disorders were administered ginseng, and the intestinal microflora, intestinal permeability and other indicators were assessed. The results showed that ginseng prevented intestinal flora disorder in mice, protected the intestinal barrier, and prevented bone loss; additionally, normal bone metabolism was maintained [182]. Puerarin is the main active component of Pueraria. OVX-induced OP rats have been used to study the mechanism through which Pueraria alleviated OP. Pueraria regulates the production of short-chain fatty acids, maintains the integrity of the intestinal flora, inhibits inflammation, and protects the intestinal barrier; thus, it plays an anti-OP role [81]. Some scholars have further studied whether quercetin protects bone by affecting the intestinal flora. An OVX-induced rat model was established, and the effects of antibiotic treatment and fecal microflora transplantation on rats were assessed. After 6 weeks of treatment, quercetin regulated the expression of short-chain fatty acids and inhibited the activation of inflammatory signals. Moreover, the intestinal flora structure of rats was enriched, the intestinal permeability of rats was protected, bone loss of rats was inhibited, and normal bone metabolism was maintained [183]. Jiangu granule is a widely used traditional Chinese medicine prescription in clinical practice. I a previous study, female SD rats were separated into a sham operation group, a Jiangugu granule group and a model group; the bilateral ovaries of the rats in the latter two groups were surgically removed. Samples were collected for analyses after 6 weeks and 12 weeks. The results revealed that Jiangu granules regulate the Gut Microbiota-Short-Chain Fatty Acids-Regulatory T Cell/T Helper 17 Cell (GM-SCFA-Treg/Th17) cell signaling axis, promote the production of short-chain fatty acids, protect the intestinal barrier and reduce bone loss [184]. Tanshinone IIA is a diterpene quinone isolated from Salvia miltiorrhiza. In C57BL/6 mice (in vivo model) and bone marrow monocytes isolated from the femoral bone marrow of C57BL/6 mice (in vitro model), tanshinone IIA stimulated the expression of the transcription factor Nuclear Factor of Activated T-cells cytoplasmic 1 (NFATc1), regulated the NF-κB and MAPK signaling pathways, reduced RANKL activity, and reduced the formation of osteoclasts [185]. Puerarin is extracted from the root of Radix Pueraria. In a study designed to explore the effects of puerarin on the proliferation, differentiation and mineralization of MC3T3-E1 osteoblasts, puerarin reduced the expression of Transient Receptor Potential Melastatin 3/microRNA-204 (TRPM3/miR-204)and promoted the growth and development of osteoblasts [186]. Paeoniflorin is the main component of peony. In Dex-induced MC3T3-E1 cells (in vitro model) and C57BL/6 mice (in vivo model) treated with paeoniflorin, changes in cell tissue, trabecular microstructure and bone turnover markers were observed; paeoniflorin increased the level of autophagy in cells, promoted the growth and development of osteoblasts, and promoted bone formation [187]. Cistanoside A is among the active ingredients of Cistanche deserticola. Primary osteoblasts were treated with 5 μM, 10 μM and 20 μM cistanoside A and then divided into groups. Then, the cells were treated with rapamycin, 3-Methyladenine (3-MA) and Dickkopf-related protein 1(Dickkopf-1), and the indices of each group were observed after culture for a period of time. Cistanoside A regulated the activation of the Wnt/β-catenin signaling pathway, further increasing the level of autophagy and promoting bone formation; the optimal dosage of cistanoside A was 10 μM [188]. Glycyrrhizin is one of the active ingredients of liquorice and has a good anti-inflammatory effect. In rats administered glycyrrhizic acid (75 mg/kg/d for 5 days; in vivo model) and rat pituitary GH3 cells (in vitro model) treated with glycyrrhizic acid, glycyrrhizic acid inhibited 11-β-hydroxy-steroid dehydrogenase in vivo and in vitro, exerted anti-inflammatory effects similar to those of glucocorticoids, and reduced the occurrence of OP [189]. In addition, Polygonum multiflorum, ginseng and knotweed have been shown to have protective effects on bones after menopause [190] (Table 2).

**Table 2:** Summary of Chinese herbal medicines for altering osteoporosis-related mechanisms

Therapeutic drugs	Research object	Mechanism	References
Andrographolide	Rats	↓Senility	[156]
Dendrobium officinale	Senility mice	↓Senility	[157]
Resveratrol	BMSCs	↓Senility ↓ROS	[159]
Emodin	OP rats	↓Inflammation	[161]
Eucommia ulmoides	OVX-OP rats	↓Inflammation	[162]
Ginsenoside	DEX-OP rats	↓Inflammation	[164]
Icariin	Mice and MC3T3-E1 cells	↓NF-κB and RANKL	[167]
Quercetin	MC3T3-E1 cells	↑Nrf2/HO-1 ↓ROS	[169]
Matrine	BMMSCs and C57BL/6-mice	↓NF-κB and ↓AKT	[171]
Polydatin	OP mice	↓MAPK	[172]
Artesunate	LPS-stimulated osteoclasts Mice	↓TLR4/TRAF6 ↓PLCγ1-Ca	[173]
Crocin	Rats	↓TNF-α and IL-6 ↓ROS	[176]
Cordycepin	BMSCs and OVX -mice	↓ROS	[177]
Gastrodin	Iron overload mice Hippocampal neurons	↑Nrf2/ARE ↓Ferroptosis	[178]
Curculigoside	MC3T3-E1 cells and mice	↓ROS and ferroptosis	[179]
QinE pill	OVX-OP rats	↑AKT/PI3K ↓Ferroptosis	[180]
Ginseng	BALB/c mice	↑Intestinal flora ↓Intestinal permeability	[182]
Puerarin	OVX-OP rats	↑Intestinal barrier ↑Short-chain fatty acid	[81]
Paeoniflorin	MC3T3-E1 cells C57BL/6-mice	↑Autophagy	[187]
Cistanoside A	OB cells	↑Autophagy	[188]
Tanshinone IIA	C57BL/6-mice BMMSCs	↑Transcription factor NFATc1	[185]
Puerarin	MC3T3-E1 cells	↓TRPM3/miR-204	[186]

↑: Increase or activation; ↓: decrease or inhibition. BMSCs bone marrow mesenchymal stem cells, ROS reactive oxygen species, OP osteoporosis, OVX-OP ovariectomy-osteoporosis, DEX-OP dexamethasone-osteoporosis, MC3T3-E1 mouse calvarial preosteoblastic cell line, subclone-E1, NF-kB nuclear factor-kappaB**,** RANKL receptor activator of nuclear factor-κB ligand, Nrf2 nuclear factor-erythroid 2-related factor 2, HO-1 heme oxygenase-1, MAPK mitogen-activated protein kinase, LPS lipopolysaccharide, TLR4 toll-like receptor 4, TRAF6 TNF receptor-associated factor 6, PLCγ1-Ca phospholipase c gamma 1-calcium, TNF-α tumor necrosis factor-alpha, IL-6 interleukin-6, PI3K phosphoinositide 3-kinase, OB osteoblasts, NFATc1 nuclear factor of activated T-cells, cytoplasmic 1, TRPM3 transient receptor potential melastatin 3, miR-204 microRNA-204

### Nonpharmacological management

Nonpharmacological management for OP includes smoking cessation, limiting alcohol and caffeine intake, and exercise, among other strategies [121]. Exercise therapy is very practical; it can increase bone density and reduce the risk of fractures; however, the choice of exercise should be individualized [191]. Exercise methods include aerobic exercise, resistance exercise, and impact training. Aerobic exercise includes jogging, swimming, and Pilates. Traditional Chinese medicines such as Taijiquan and Baduanjin also include aerobic exercise. Resistance exercise includes weightlifting, push-ups, and impact training, such as skipping rope [192, 193]. Research has shown that for postmenopausal women with OP, regular light to moderate walking exercise can decrease physical pain, increase walking speed, enhance physical balance and increase daily activity ability [194]. Compared with high-intensity interval training or vitamin D alone, the combined treatment of high-intensity interval training and vitamin D supplements significantly increases bone mineral density in OP patients [195]. Another study revealed that exercise alleviates OP by regulating the secretion of the senescence-associated secretory phenotype [196]. Research has shown that ultraviolet therapy and extracorporeal shock wave therapy can increase bone mass in OP patients. Microwave therapy and medium-frequency or low-frequency pulses have good pain-relieving effects in OP patients [197].

### Emerging therapeutic strategies

**Fig. 5:**
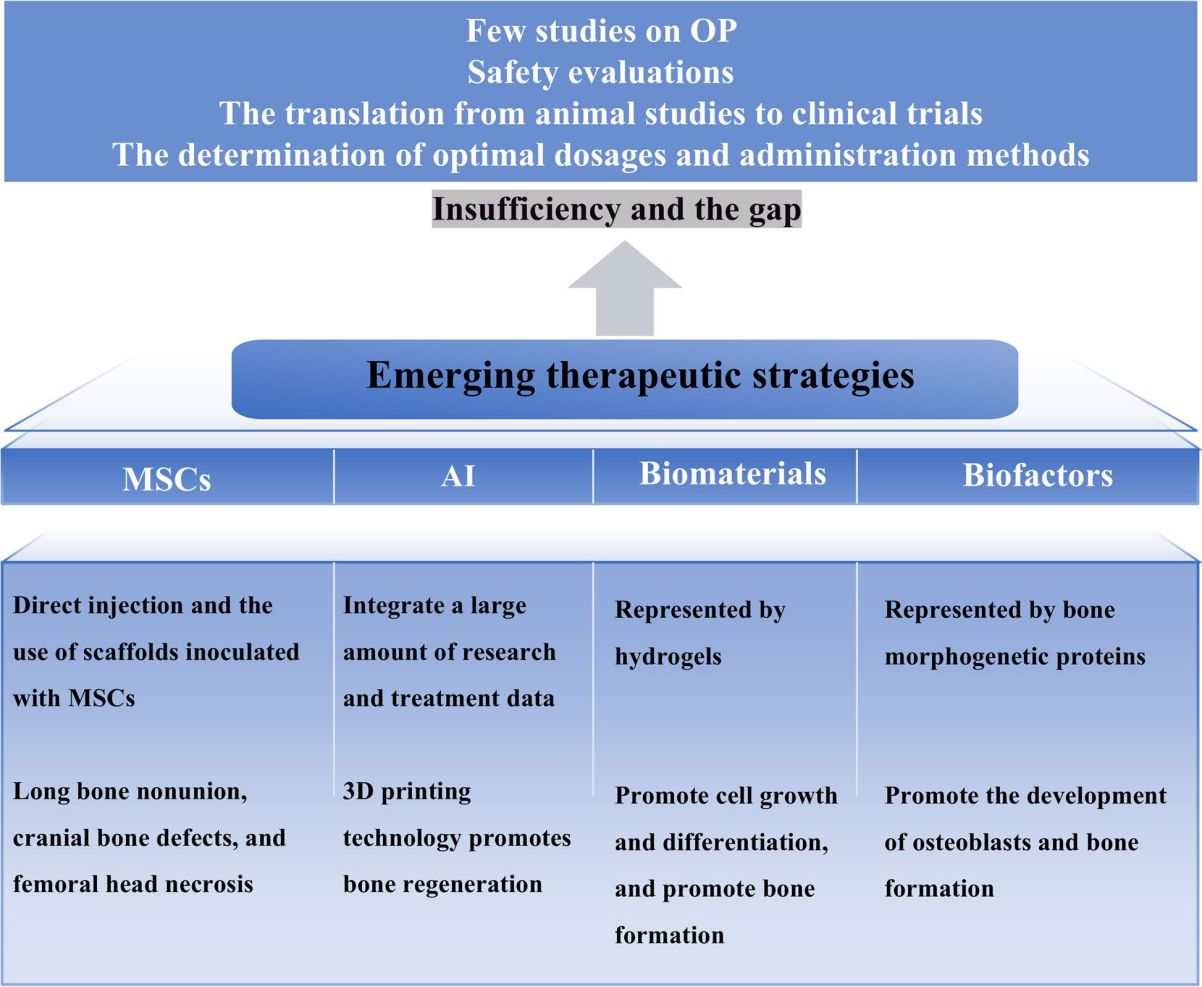
Emerging treatment options and their related shortcomings. There are four emerging treatment options: MSCs, AI, biomaterials, and biofactors. There is still a significant gap between emerging treatment options and clinical application, for example, few studies on OP, the translation from animal studies to clinical trials, the determination of optimal dosages and administration methods, and safety evaluations. MSCs marrow mesenchymal stem cells, AI artificial intelligence, OP osteoporosis, 3D three – dimensional

Mesenchymal stem cells (MSCs) can differentiate into osteoblasts and play important roles in bone formation. MSCs are derived mainly from the bone marrow but are present in other tissues of the body [198]. MSCs have many functions. First, self-renewal is a very important function for bone regeneration. MSCs undergo mitosis and self-renew, maintain an undifferentiated state, and promote tissue regeneration [199]. Second, MSCs can regulate immune factors and inflammatory factors, inhibit the development of inflammation, and increase tissue healin levels [200–202]. The above characteristics have led to widespread attention being given to MSCs and their treatment effects on various disease. Many clinical trials of MSCs for the treatment of orthopedic diseases have been conducted. Intervention techniques include direct injection and the use of scaffolds inoculated with MSCs. In the context of orthopedic diseases such as long bone nonunion, cranial bone defects, and femoral head necrosis, MSCs play a therapeutic role [203]. At present, there are no clinical studies of MSCs specifically targeting OP. However, on the basis of existing research, MSCs are potential targets for the treatment of OP. In addition, research has shown that biofactors, such as bone morphogenetic proteins, can promote the activity of osteoblasts and further enhance bone formation. Biomaterials such as hydrogels can provide favorable conditions for cell growth and differentiation, thereby promoting bone generation. Biofactors and biomaterials offer new directions for the treatment of OP [204, 205]. With the development of technology, artificial intelligence has been applied to health care. With respect to orthopedic diseases, artificial intelligence has promoted the development of biomaterials related to bone regeneration [206–208]. Artificial intelligence can integrate a large amount of research data and treatment data to provide reference data for the development of orthopedic disease plans. In addition, Three-Dimensional (3D) printing has been applied to bone regeneration [203, 209]. Artificial intelligence can also play various auxiliary roles in the treatment of OP. In the future, more related research will be conducted to further promote the treatment of OP (Fig. 5).

In summary, each of the four treatment methods has its own advantages and potential. Western medicine treatment is the most well developed and still occupies a dominant position. Some proprietary Chinese medicines have also been put into clinical practice and have achieved good clinical results. Nonpharmacological treatment is very practical and can be used as an auxiliary treatment method. Emerging treatment methods provide new ideas for the treatment of OP.

## Conclusion and perspective

**Fig. 6:**
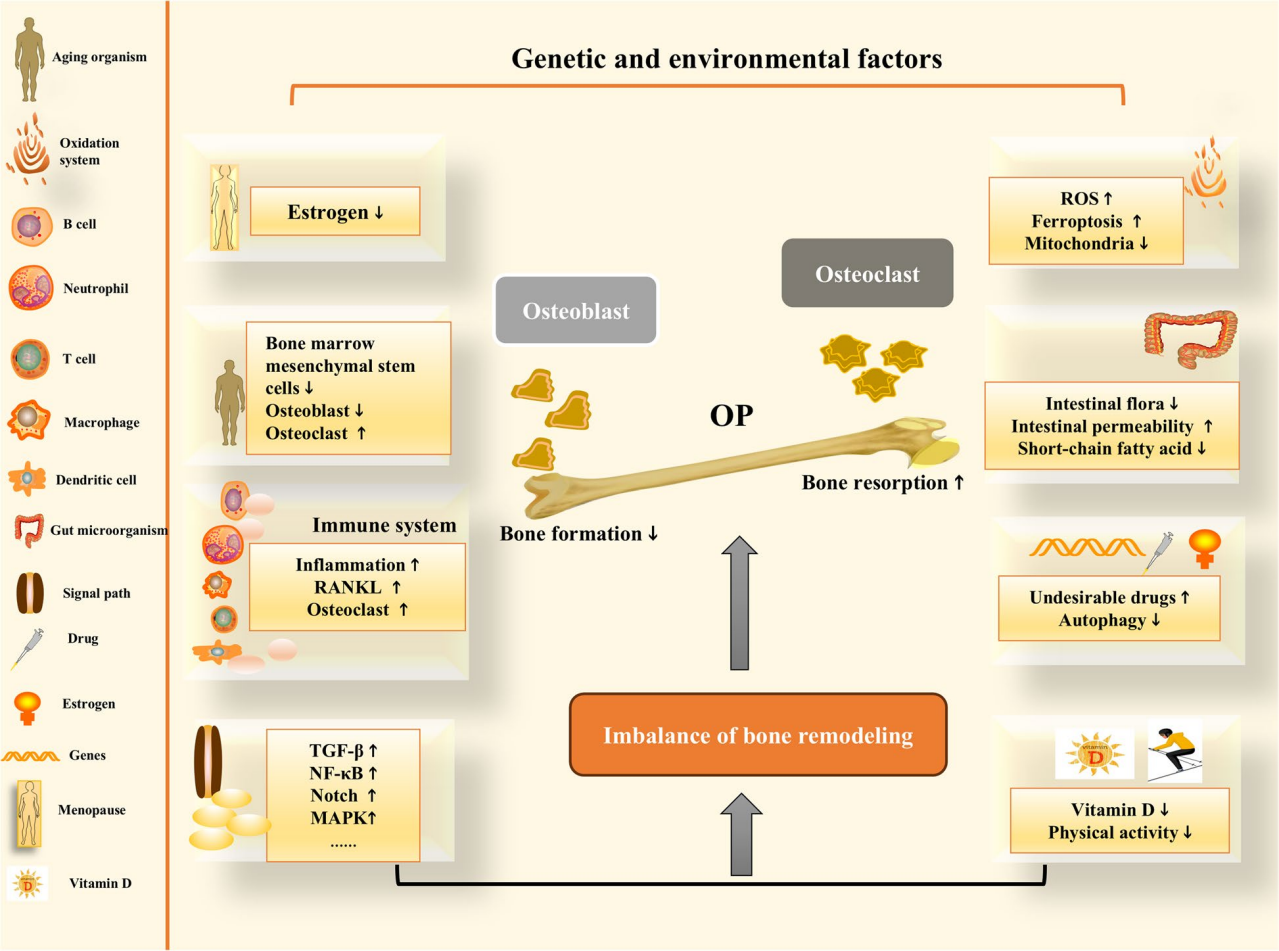
The molecular mechanism of osteoporosis (OP). Osteoporosis results from the combined action of genetic and environmental factors. Various factors cause increased bone resorption and decreased bone formation, leading to an imbalance in bone remodeling, reduced bone mass, and ultimately osteoporosis. These factors include aging, abnormal immune system activation, inflammatory responses, abnormal signaling pathway regulation, intestinal microbiota status, oxidative stress, autophagy, vitamin D deficiency, and reduced physical activity. Estrogen deficiency in postmenopausal women is also an important cause of osteoporosis. OP osteoporosis, RANKL receptor activator of nuclear factor kappa-B ligand, ROS reactive oxygen species, NF-κB nuclear factor kappa-B, MAPK mitogen-activated protein kinase,TGF-β transforming growth factor-β

OP can clinically manifest as pain, spinal deformation, and osteoporotic fracture, among other symptoms, and is a global bone health problem. If OP is not treated in a timely manner, lung infection, lower limb vein thrombosis and other complications after long-term bed rest can occur, severely affecting health and quality of life. The pathogenesis and influencing factors of OP are complex and include a series of factors, such as natural aging, genes, the immune system, inflammation, oxidative stress, iron metabolism, and intestinal microbes. Secondary factors include postmenopausal status and the use of certain drugs. Multiple factors can affect each other, and therefore, the underlying mechanism is intricate (Fig. 6). Studies on the prevention and treatment of OP have attracted the attention of scholars worldwide, and numerous relevant studies, including in vivo and in vitro studies, have emerged. At present, many drugs and methods for the treatment of OP have emerged clinically, and remarkable research progress has been made. This paper systematically introduces the molecular mechanism of OP formation, as well as progress in OP diagnosis and therapeutic interventions.

In terms of treatment, this paper elaborates on Western medicine treatment, traditional Chinese medicine treatment, nonpharmacological management, and emerging treatment strategies. There are a wide variety of drugs used for Western medicine treatment. In addition to traditional antiresorptive drugs, abaloparatide, teriparatide, and romosozumab provide more options for OP patients and have shown good therapeutic effects in clinical practice. At present, Western medicine treatment still occupies an absolute dominant position, and related experimental research is the most standardized and complete.

Chinese herbal medicines are rich in variety, offering a wide range of choices, and have a long history; they also show good research prospects for the treatment of OP. Research on the treatment of OP with Chinese herbal medicines, including single Chinese herbs, Chinese patent medicines, and compound preparations, is needed. With respect to the influencing factors of OP, there are corresponding research reports on traditional Chinese medicine, indicating a wide coverage of related research. Moreover, research on traditional Chinese medicine for each influencing factor has yielded good results. There are already proprietary Chinese medicine preparations approved for the clinical treatment of OP, reflecting the definite efficacy and great potential of traditional Chinese medicine in the treatment of OP. In addition, compared with Western medicines for treating OP, Chinese herbal medicines have fewer contraindications. Currently, the reported adverse reactions are mostly gastrointestinal discomfort, and no severe adverse reactions have occurred, indicating high safety. Compared with studies on influencing factors such as oxidative stress, immune factors, and inflammation, research on traditional Chinese medicine in terms of autophagy, genes, and aging is relatively weak. Research on traditional Chinese medicine compound preparations has focused mostly on overall therapeutic effects, lacking in-depth studies of specific drugs. Only a few studies have conducted dose comparisons, and almost no studies have compared administration methods. Most of the research is still in the experimental model stage, and therefore, there is still a long way to go before clinical application. Chinese herbal medicine research must seek to overcome challenges such as bioavailability, pharmacokinetics, and dosage optimization; lead to high-quality clinical research; and actively transition from animal models to clinical application. Jintiange capsules, which mainly contain artificial tiger bone powder, can be taken as an example. On the basis of animal experiments, randomized, double-blind, double-dummy, positive-controlled, multicenter clinical trials have successfully verified the therapeutic effect of Jintiange capsules against primary osteoporosis, confirming the therapeutic effect of Chinese herbal medicines at a clear therapeutic dosage and with rigorous research methods. Jintiange capsules have been put into clinical use [210]. Nonpharmacological management includes exercise and physical therapy; however, the choice and intensity need to be individualized, and these treatments can only be used as adjuvant options. The rise of stem cell therapy, artificial intelligence, biomaterials, etc., has provided new directions and therapeutic targets for the treatment of OP. However, large gaps in knowledge must be addressed before emerging treatment methods can be applied in clinical practice.

In summary, significant progress has been made in research on the pathogenesis and treatment of OP. However, the prevention and treatment of OP remain a global challenge. The pathological mechanism of OP is complex and involves various factors, such as genes, inflammation, immunity, and oxidative stress. Drugs for Western medicine treatment are being developed rapidly. Emerging drugs, such as drugs that promote bone formation and that regulate both bone resorption and bone formation, have been put into clinical use and have achieved good clinical efficacy. In terms of traditional Chinese medicine, only Chinese patent medicine preparations have been put into clinical use. Most other related studies have progressed only to the stage of animal or experimental models, lacking comparisons of drug dosages and administration methods. Bioavailability and dosage optimization are challenges that must be overcome prior to studies on Chinese herbal medicines transitioning from basic research to clinical research. Well-designed studies will help research related to traditional Chinese medicine gain global recognition. Emerging treatment methods such as stem cell therapy, artificial intelligence, and biomaterials have been applied to treat orthopedic diseases. However, research related to OP is lacking, and new directions for OP treatment are needed in the future.

## Data Availability

Not applicable.
